# Effects of toxic *Microcystis aeruginosa* on the silver carp *Hypophthalmichtys molitrix* revealed by hepatic RNA-seq and miRNA-seq

**DOI:** 10.1038/s41598-017-10335-9

**Published:** 2017-09-05

**Authors:** Menghong Hu, Xiancheng Qu, Lisha Pan, Chunxue Fu, Peixuan Jia, Qigen Liu, Youji Wang

**Affiliations:** 1 National Demonstration Center for Experimental Fisheries Science Education, Shanghai Ocean University, Shanghai, 201306 China; 2 The Key Laboratory of Exploration and Utilization of Aquatic Genetic Resources, Ministry of Education, Shanghai, 201306 China; 3 International Research Center for Marine Biosciences at Shanghai Ocean University, Ministry of Science and Technology, Shanghai, China; 4 Centre for Research on Environmental Ecology and Fish Nutrion (CREEFN) of the Ministry Agriculture, Shanghai Ocean University, Shanghai, China; 5 Key Laboratory of Freshwater Aquatic Genetic Resources, Ministry of Agriculture Ministry, Ocean University, Shanghai, China

## Abstract

High-throughput sequencing was applied to analyze the effects of toxic *Microcystis aeruginosa* on the silver carp *Hypophthalmichthys molitrix*. Silver carps were exposed to two cyanobacteria species (toxic and non-toxic) for RNA-seq and miRNA-seq analysis. RNA-seq revealed that the liver tissue contained 105,379 unigenes. Of these genes, 143 were significantly differentiated, 82 were markedly up-regulated, and 61 were remarkably down-regulated. GO term enrichment analysis indicated that 35 of the 154 enriched GO terms were significantly enriched. KEGG pathway enrichment analysis demonstrated that 17 of the 118 enriched KEGG pathways were significantly enriched. A considerable number of disease/immune-associated GO terms and significantly enriched KEGG pathways were also observed. The sequence length determined by miRNA-seq was mainly distributed in 20–23 bp and composed of 882,620 unique small RNAs, and 53% of these RNAs were annotated to miRNAs. As confirmed, 272 known miRNAs were differentially expressed, 453 novel miRNAs were predicted, 112 miRNAs were well matched with 7,623 target genes, and 203 novel miRNAs were matched with 15,453 target genes. qPCR also indicated that *Steap4*, *Cyp7a1*, *CABZ01088134.1*, and *PPP1R3G* were significantly differentially expressed and might play major roles in the toxic, detoxifying, and antitoxic mechanisms of microcystin in fish.

## Introduction

With the exacerbating eutrophication of inland waters, the frequency and intensity of the outbreak of freshwater algal blooms have become increasingly serious. Under the condition, ruptured toxic cyanobacterial cells often release algal toxins, especially microcystin (MC), which is the most common and most harmful^1–4^. MC is a hepatotoxic intracellular endotoxin synthesized in cells. Once algal cells disintegrate, endotoxins are released and thus cause water quality deterioration and harmful effects on aquatic animals, plants, and human health^5, 6^. Beasley *et al*. (2000) found that MC mainly acts on the liver by inducing increased liver weight index, liver tissue swelling and a series of enzymatic changes that may damage the liver cell skeleton and cause hepatic hemorrhage and necrosis^7^. Yu *et al*. (2001) reflected that the presence of MC in drinking water is positively correlated with the incidence of primary hepatic carcinoma and colorectal carcinoma^8^. Alverca *et al*. (2009) revealed that toxic algal contamination threatened a small town in Pennsylvania in the USA in 1975 and induced acute gastroenteritis on approximately 8,000 people, which accounted for 62% of the local population^9^. In 1996, a hemodialysis center of 130 patients in Brazil had 117 cases with abnormal symptoms because of dialysates contaminated with cyanobacterial toxins, and more than 50 affected individuals died^10^. Yu (1995) and Chen *et al*. (1996) pointed out that algal toxins have also been detected in different sources of drinking water in China, and results indicate that the presence of MC in drinking water from ponds and rivers is a risk factor of the high incidence of live cancer in southeast coastal areas^11, 12^. Chen *et al*. (2009) disclosed that MCs detected from blood specimens of professional fishermen from Chaohu in 2005, implying that long-term chronic exposure to MC causes some degree of liver injury^13^. Magalhaes *et al*. (2001; 2003) presented that the bioaccumulation of MC in aquatic animals subjected to long-term exposure to MC likely causes harmful effects on human health through food chains^14, 15^. Several epidemiological studies have shown that MCs cause harmful effects on human health^1–3^. Wozny *et al*. (2016) showed clearly biphasic effects of MC exposure on fish liver^16^.

MC is a class of monocyclic heptapeptide compounds with biological activity. Ge *et al*. (2009) contended that more than 100 kinds of MC isomers have been discovered^5, 17^. Alverca *et al*. (2009) showed that the most prevalent and toxic are MC-LR, MC-RR, and MC-YR^9^. MC toxicity is highly specific to its target organ - the liver^18^. The toxicity mechanism of MC in the liver mainly involves four aspects: inhibiting the activity of proteinphosphatase 1/2 A (PP1/2 A)^19, 20^; inducing cellular oxidative stress, namely, the over-expressions of substances with oxidative activity^21, 22^; inducing DNA damages^23, 24^; and causing disturbance of metabolism of lipids in liver^25^. Upon penetrating the fish body, MC may undergo a non-synthetic transformation (I-phase reaction) with monooxygenases, such as cytochrome P450, as a catalyst^26–28^. MC or its metabolites may produce an addition reaction (II-phase reaction) with endogenous ligands^26–28^. Pflugmacher (2016) reviewed that phase I enzymes are not directly involved in the biotransformation of microcystins, but their activation may explain the increase in ROS generation in the liver and therefore contributing to the oxidative stress generated by microcystins^29^. Current studies on the detoxifying mechanism of MC in fish have focused on the addition reaction (II-phase reaction) of MC with glutathione (GSH) as catalyzed by GST^26–28, 30^. However, MC-detoxifying genes in fish and their up-regulation mechanisms have yet to be further examined^26–28^.

RNA-seq is based on high-throughput sequencing and applied to detecting almost all transcriptional sequences of target sepcies at a certain state. With its high-throughput sequencing capability, high sensitivity, wide range, short analysis time, high efficiency, high reproducibility, and low cost, the technique has been developed rapidly and has been widely used in transcriptome analysis^31, 32^. Thus, RNA-seq, which is independent of genomic sequence information, is of great value to non-model organisms with unknown genome sequence information and is commonly employed in transcriptome studies of various plants and animals. Harke and Gobler (2013) evaluated the transcriptional response of toxic *M. aeruginosa* in a particular growth process by RNA-Seq and provided a thorough understanding of the nutrition physiology of cyanobacterial toxins^33^. A total of 218 KEGG pathways have also been determined through RNA-seq, and the positive selection of five genes involved in glutathione biosynthesis in zebrafish and silver carps has been identified^34^.

Various kinds of small RNAs in non-coding RNAs constitute a highly complex RNA regulatory network in cells and play important roles in regulating all life activities such as cell proliferation and differentiation, ontogenesis, antiviral mechanisms, and tumorigenesis. miRNA is a group of non-coding RNAs. Lee *et al*. (1993) first discovered it in *Caenorhabditis elegans* ^35^. It has a unit length of approximately 22 bp and is formed by a series of RNase III endonucleases, transporters, and other enzymes. Hock and Meister (2008) instructed the miRNA complex (RNA-induced silencing complex, *RISC*) to cleave or translate the target mRNA by complementary pairing with the 3ʹ-UTR of the target gene^36^. High-throughput sequencing has been gradually applied as a new technique to mine miRNA, directly reveal the expression of a differentially expressed miRNA, detect novel or species-specific miRNAs, and predict their target genes. Molecular evidences show that microcystin-LR (MC-LR) exposure causes perturbations of microRNA (miRNA) signaling in fish^37^. The roles of 6 differentially expressed miRNAs in the liver tissues of *Coregonus lavaretus* intraperitoneally injected with MC were clarified^38^. A total of 166 conservative miRNAs and 22 novel miRNAs were detected in silver carps by high-throughput sequencing, and the loss and gain of several members of the miRNA family suggested the possible evolutionary process of miRNA replication in animals^39^.

Silver carps are filter-feeding fish that feed mainly on planktonic organic debris in water. They eat algae in large quantities and survive for a long time in water where blooms occur. The phenomenon is the basis for the biological control of harmful algae with silver carps. A LD50 of 350 μg/kg in silver carps injected intraperitoneally with MC-LR indicates a strong tolerance to MC^40^. The most-studied detoxification enzymes in fish exposed to MCs are the glutathione-S-transferases (GSTs). After MC absorption by fish, fish detoxify the MC by the addition reaction between MC and GSH by using GST as a catalyst. Thus GST expression can be considered as an ideal indicator for MC detoxification in fish liver^41, 42^. In the present study, the first step of detoxifying the fish body from MC for reference and the time point when GST began to produce effects on the liver tissues of silver carps obtained from the two kinds of water samples were determined. During the toxic *M. aeruginosa* exposure, some disease/immune-involved pathways are expected to play important roles in the detoxifying process. To understand the toxic effects of cyanobacteria on freshwater fish, the transcriptomic responses of the silver carp exposed to microcystin-producing cyanobacteria were investigated by using combination of RNA-seq and miRNA-seq. The effects of MC on the temporal and spatial expressions of genes in the liver tissues of silver carps were comprehensively analyzed through bioinformatics. The toxic, detoxifying, and antitoxic mechanisms of MC in silver carps were also illustrated by analyzing four relevant key genes, i.e., Metalloreductase STEAP4 (*steap4*), Cholesterol 7-alpha-monooxygenase (*Cyp7a1*), Protein phosphatase 1 regulatory subunit 3G-like (*PPP1R3G*) and *trans*-2-enoyl-CoA reductase, mitochondrial-like (*CABZ01088134.1*), which may play important roles in molecular responses to MC.

## Results

### Toxin assay on *M. aeruginosa*

The toxin types and contents of the two types of *M. aeruginosa* were determined by HPLC-MS. Results show that in toxic *M. aeruginosa* (1.3 × 10^8^ cell/L), MC took the form of MC-LR, instead of any other type, with toxin content of 35ng/mL, whereas non-toxic *M. aeruginosa* (1.3 × 10^8^ cell/L) did not contain MC.

### Expression of GST by RT-PCR

**Figure 1:**
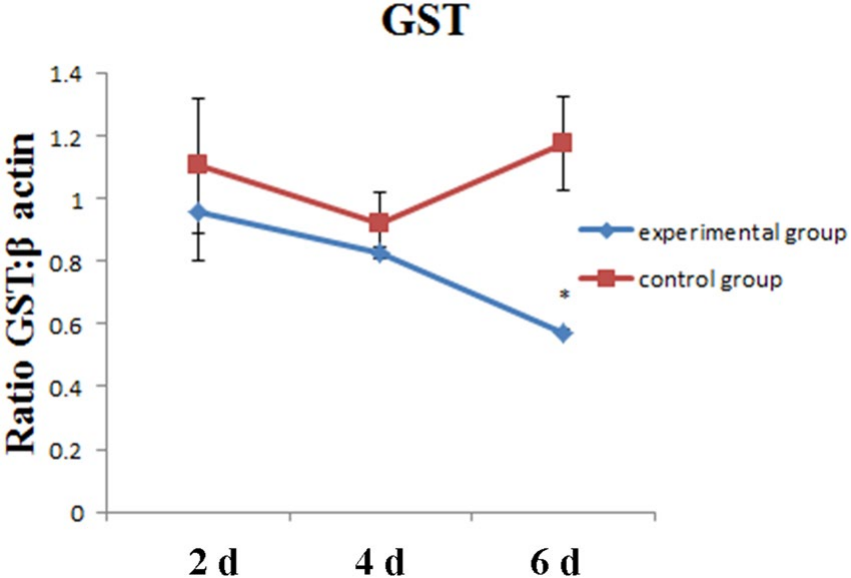
Relative expression level of GST gene in the liver of silver carp breeding in the two kinds of waters (* denotes significant differences between experimental and control groups, P < 0.05).

With β-actin as the reference gene, the relative expression of GST in liver tissues from silver carps feeding for 2, 4, and 6 d in the two kinds of water bodies (1.3 × 10^8^ cell/L) was detected by RT-PCR. The results show that compared with that in the control group, the relative GST expression in the experimental group was markedly reduced on 6 d (P < 0.05) (Fig. 1), indicating that at the time point, GST began to play its roles in the liver tissues and react with MC. As such, 6 d was used as the time point at which the liver tissue specimens from silver carps of the experimental and control groups were subjected to RNA-seq and miRNA-seq.

### RNA-SeQC and de-novo assembly

RNA-seq was determined using Illumina HiSeq. 4000. Experimental and control groups produced 105,599,760 and 113,986,118 raw reads, respectively. After quality control and screening, the experimental and control groups were streamlined into 103,677,062 and 111,469,790 clean reads, respectively. Furthermore, 105,379 unigenes (average length: 693.65 bp, N50: 1269 bp, and proportion of GC: 43.17) and 129,559 transcripts (average length: 831.01 bp, N50: 1664 bp, and proportion of GC: 43.88) were obtained after de-novo assembly using Trinity. The sequence lengths of 82.95% unigenes and those of 77.36% transcripts were less than 1,000 bp.

### Gene annotations

**Figure 2:**
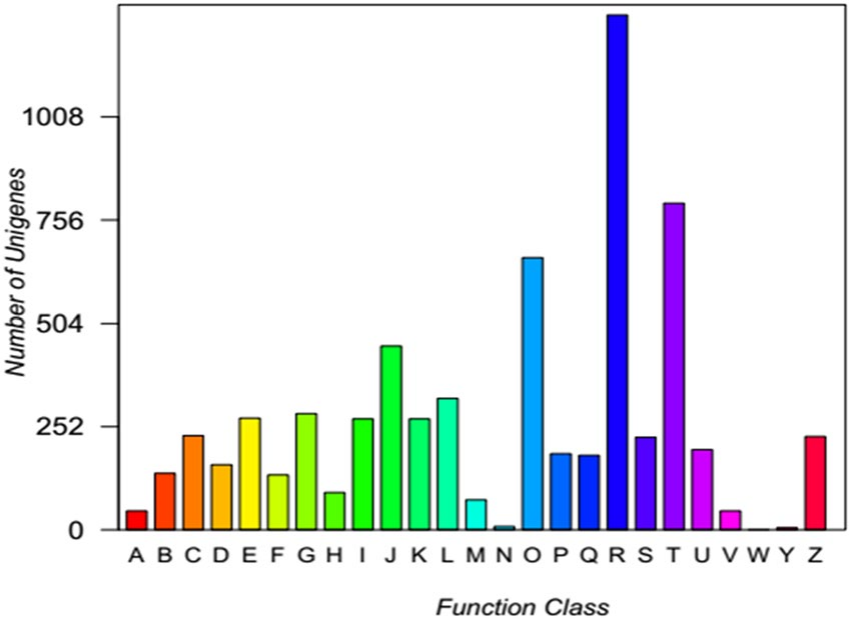
COG function classification. A, RNA processing and modification; B, Chromatin structure and dynamics; C, Energy production and conversion; D, Cell cycle control, cell division, and chromosome partitioning; E, Amino acid transport and metabolism; F, Nucleotide transport and metabolism; G, Carbohydrate transport and metabolism; H, Coenzyme transport and metabolism; I, Lipid transport and metabolism; J, Translation, ribosomal structure, and biogenesis; K, Transcription; L, Replication, recombination, and repair; M, Cell wall/membrane/envelope biogenesis; N, Cell motility; O, Posttranslational modification, protein turnover, and chaperones; P, Inorganic ion transport and metabolism; Q, Secondary metabolites biosynthesis, transport, and catabolism; R, General function prediction only; S, Function unknown; T, Signal transduction mechanisms; U, Intracellular trafficking, secretion, and vesicular transport; V, Defense mechanisms; W, Extracellular structures; Y, Nuclear structure; and Z, Cytoskeleton.

Of the genes obtained by RNA-seq, a total of 6,535 genes were annotated to 25 COG terms (Fig. 2) corresponding to the following types: ABJKL, belonging to information storage and processing, DMNOTUVWYZ, belonging to cellular processes and signaling, CEFGHIPQ, belonging to metabolism, and lastly, RS, belonging to poorly characterized. The most annotated types of genes were general function prediction only (1,257), signal transduction mechanisms (797), and posttranslational modification, protein turnover, chaperones (664).

**Figure 3:**
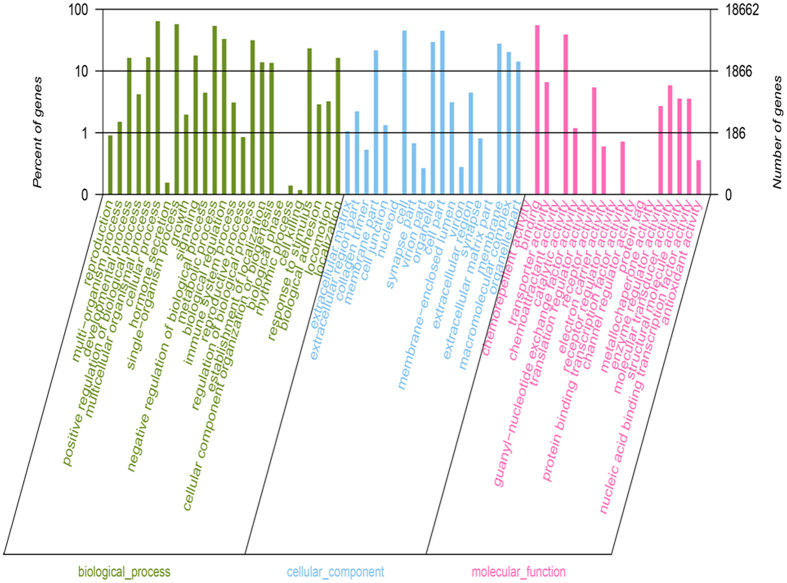
GO Function Classification.

In the sequencing, 18,662 genes were annotated to three primary GO terms, namely, biological process, cellular component, and molecular function. Figure 3 shows 25 secondary GO terms of biological process, including cellular process, single organism process, metabolic process and biological regulation; 19 cellular component secondary GO terms, including membrane part, cell, organelle and cell part; and 19 secondary GO terms of molecular function, including binding, transporter activity and catalytic activity. The 5 most inclusive gene types were cellular process (11,982 genes, accounting to 64.21%, GO:0009987), single organism process (10,728 genes, 57.49%, GO:0044699), binding (10,293 genes, 55.15%, GO:0005488), metabolic process (10,045 genes, 53.83%, GO:0008152), and single organism cellular (9,107 genes, 48.80%, GO:0044763).

**Figure 4:**
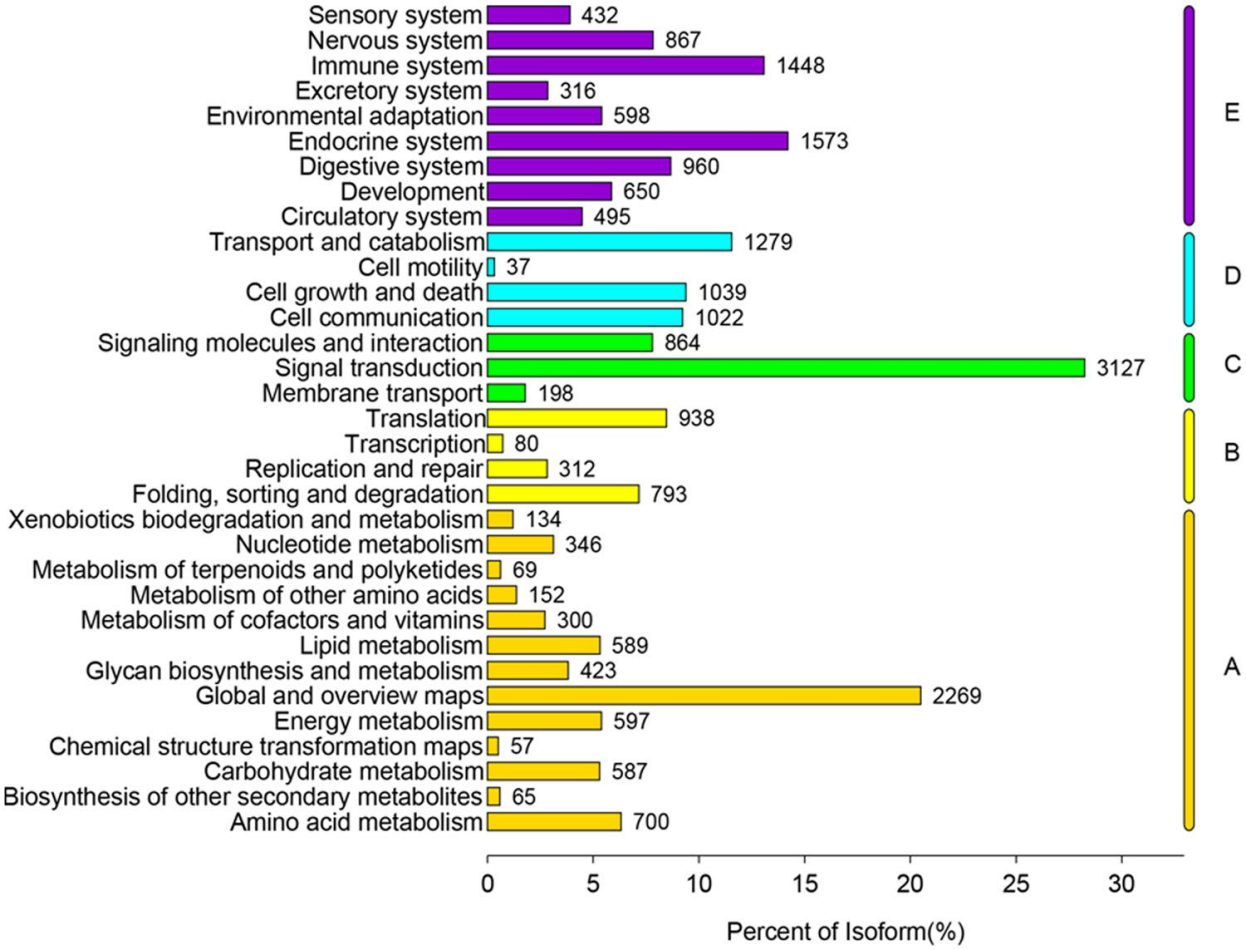
KEGG Pathway Classification.

A total of 49,018 genes were involved in 348 KEGG pathways. The 20 most active pathways included some very important ones, such as pathways in cancer, PI3K−Akt signaling pathway, focal adhesion, proteoglycans in cancer, HTLV−I infection. Of these pathways, 9 corresponded to signal pathways and 7 were disease-associated pathways. The genes were divided into five branches based on KO annotations by the KEGG pathways: A. metabolism; B. genetic information processing; C. environmental information processing; D. cellular processes; and E. organismal systems. The five branches were subdivided into 33 small branches, wherein the 2 most inclusive gene types were signal transduction (3,127) and global and overview maps (2,269) (Fig. 4).

### Analysis on DEGs

A total of 35,371 differentially expressed genes were detected in the experimental and control groups. Of these genes, 13,314 were up-regulated, 22,057 were down-regulated, 143 were significantly differentiated, 82 were markedly up-regulated, and 61 were remarkably down-regulated genes. Moreover, 67 genes were expressed in the control group but not in the experimental group, whereas 42 genes were expressed in the experimental group but not in the control group.

Through the GO term enrichment analysis on DEGs, 154 enriched GO terms were determined, 35 of which were significantly enriched (P < 0.05). Some biological processes included multicellular organismal catabolic process, multicellular organismal macromolecule metabolic process, collagen metabolic process and multicellular organismal metabolic process. Cellular components contained MHC protein complex, MHC class I protein complex, immunoglobulin complex, and endoplasmic reticulum lumen. Molecular function included interleukin 1 receptor binding, peptidase activity, L amino acid peptide activity, and hydroxymethylglutarylCoA reductase (*NADPH*) activity. A large number of DEGs were also involved in the GO terms of binding, cellular process, metabolic process, single organism process and catalytic activity. Moreover, considerable immune-associated GO terms and various metabolism-associated GO terms were significantly enriched such as regulation of immune response, response to external stimulus, regulation of immune system process, collagen catabolic process, multicellular organismal catabolic process, and multicellular organismal macromolecule metabolic process.

Based on the KEGG enrichment analysis of DEGs, 17 of the 118 enriched KEGG pathways, including cell adhesion molecules (*CAMs*), protein processing in endoplasmic reticulum, and antigen processing and presentation, were significantly enriched (P < 0.05). Other enriched pathways were also associated with diseases and immunity, such as viral myocarditis, type I diabetes mellitus, and autoimmune thyroid disease.

### RNA-SeQC and annotations

The experimental and control groups had 25,479,021 and 28,176,747 raw reads, respectively. Through quality control and filtration, the experimental and control groups respectively reduced to 20,888,695 and 227,000,005 clean reads with a length of 18–32 bp. Sequences with a length of 20–23 bp were densely distributed. The small RNAs obtained through Blast were annotated to Rfam library, where non-miRNA sequences, such as rRNA, scRNA, snoRNA, snRNA, and tRNA, were eliminated. After 882,620 reads of all the specimens were compared with the Rfam library, miRNAs accounted for 53% of the total Unique sequences, in which 272 differentially expressed known miRNAs were obtained by sequencing and 453 novel miRNAs were predicted. Based on the orthologous gene classification and comparison of miRNA target genes with DEGs in the RNA-seq data, 105 known miRNAs matched 163 differentially expressed RNAs, while 170 novel miRNAs matched 163 DEGs.

### Expression of known and novel miRNAs and prediction of their target genes

After sequencing was performed, 272 known miRNAs were differentially expressed. Of these miRNAs, 119 were up-regulated genes (including 17 markedly up-regulated) and 153 were down-regulated (36 remarkably down-regulated). Furthermore, 453 were novel differentially expressed miRNAs. Of these genes, 221 were up-regulated (62 were markedly up-regulated) and 200 down-regulated (53 were remarkably down-regulated). A total of 112 known miRNAs matched 7,623 target genes well, and 203 novel miRNAs matched 15,453 target genes well.

### Conjoint analysis on RNA-seq and miRNA-seq data and fluorescence quantitative PCR

The conjoint analysis on miRNA and RNA data revealed that 105 known miRNAs matched 163 differentially expressed RNAs, and 730 readings were produced. When known miRNAs were down-regulated, 99 DEGs were up-regulated, involving 38 different miRNAs and 54 DEGs. By contrast, when known miRNAs were up-regulated, 130 DEGs were down-regulated, which involved 39 different miRNAs and 57 DEGs. Furthermore, 170 novel miRNAs matched 163 DEGs and thus formed 1,198 readings. As novel miRNAs were down-regulated, 249 DEGs, including 60 different novel miRNAs and 81 DEGs, were up-regulated. By contrast, as novel miRNAs were up-regulated, 157 DEGs, including 63 novel miRNAs and 48 DEGs, were down-regulated.

In the present study, after the conjoint analysis on pre-RNA and miRNA data, four candidate genes, namely *CABZ01088134.1*, *steap4*, *Cyp7a1*, and *PPP1R3G*, which may play critical roles in the intoxicating, detoxifying, and antitoxic mechanisms of MC in fish, were preliminarily selected. By qPCR with β-actin as the reference gene, the roles of those genes in the liver tissues of silver carps under the presence of MC were examined on 2, 4, and 6 d, and the results were validated with the RNA-seq data of the specimens on 6 d.

**Figure 5:**
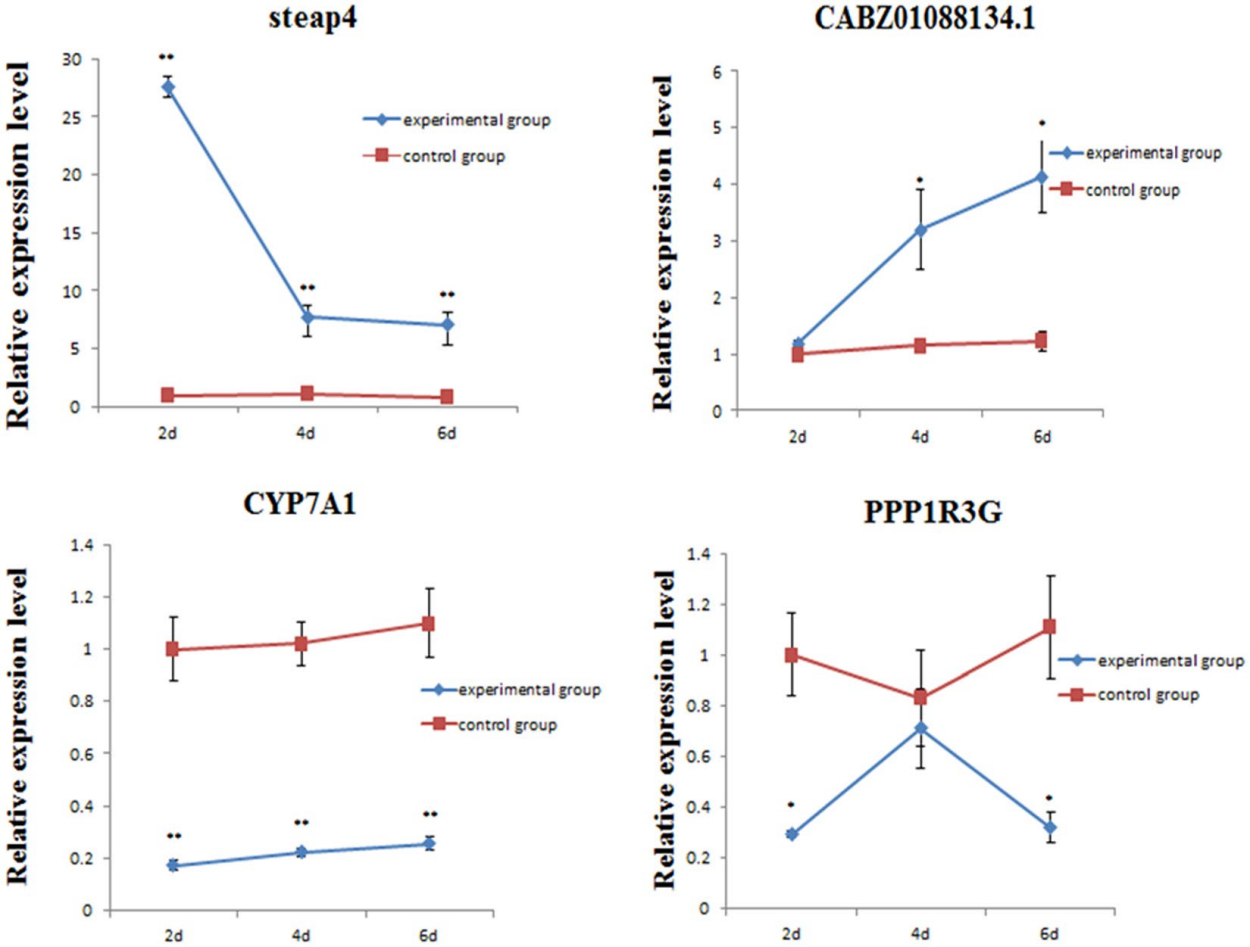
Expression levels of *Steap4*, *CABZ01088134.1*, *Cyp7a1*, and *PPP1R3G* in the silver craps feeding for 2, 4 and 6 d in the two kinds of waters (*P < 0.05, **P < 0.01).

Figure 5 shows the expression levels of the four genes at the three time points. Compared with that in the control group, the expression of *steap4* in the experimental group was markedly up-regulated and slightly increased on 4 and 6 d. Its expression significantly changed and maintained high levels. The expression of *CABZ01088134.1* was up-regulated with the increasing action time of MC, and significant changes were observed at 4 and 6 d. The expression of *Cyp7a1* was remarkably down-regulated since 2 d. Thereafter, its expression was maintained at a low level. The relative expression of *PPP1R3G* was remarkably down-regulated at 2 and 6 d, and the relative expression at 4 d was higher than that at 2 d. Conversely, its expression was lower than that in the control group.

**Figure 6:**
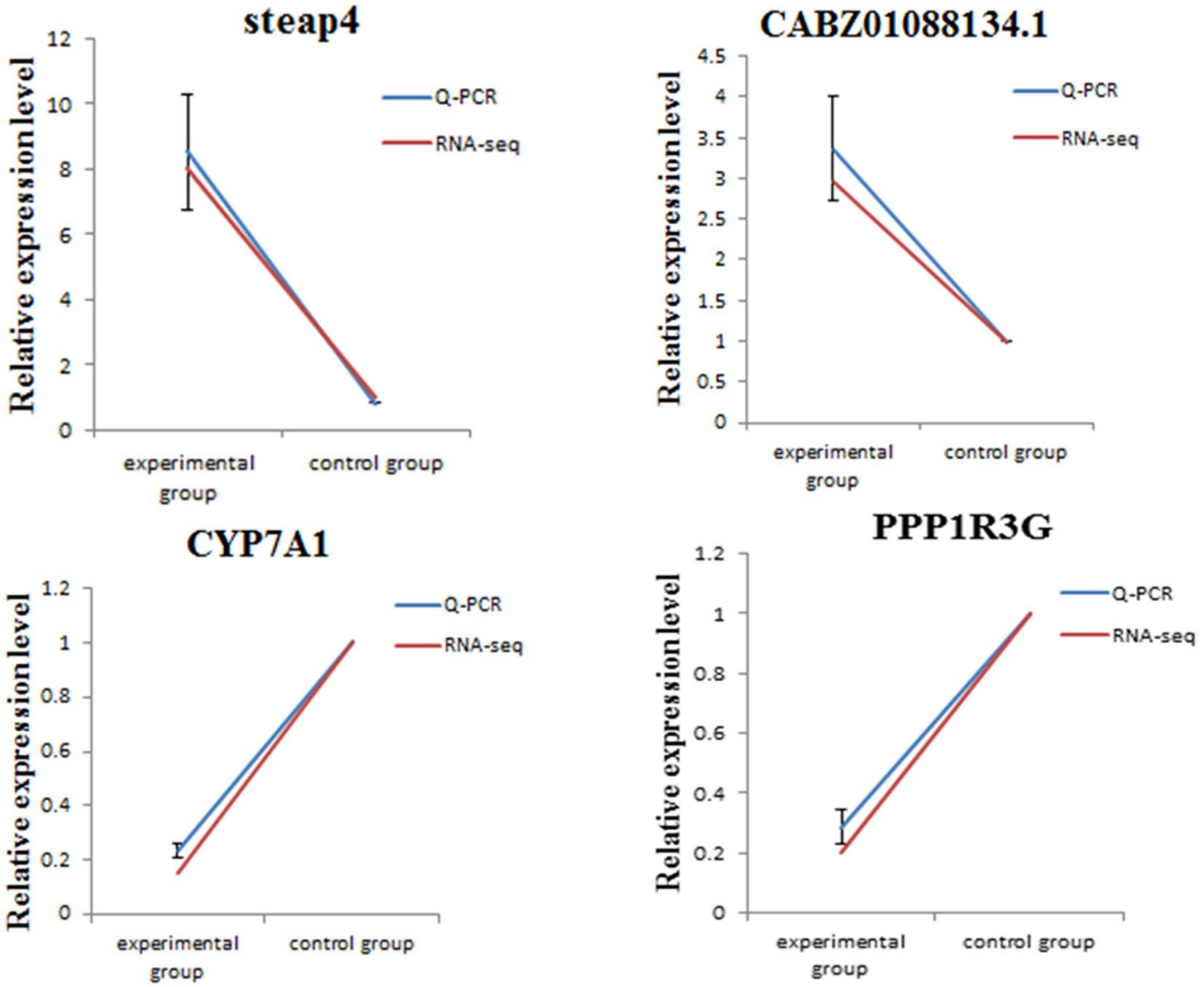
Comparative analysis of mRNA-seq and qPCR for *Steap4*, *CABZ01088134.1*, *Cyp7a1* and *PPP1R3G*.

Figure 6 shows the change trends of the RNA and qPCR data between the experimental and control groups at 6 d. Compared with the control group, the relative expressions of *steap4* and *CABZ01088134.1* in the experimental group were up-regulated, whereas the relative expressions of *Cyp7a1* and *PPP1R3G* were down-regulated. The RNA results were consistent with the qPCR findings (Fig. 6). Accordingly, the reliability and accuracy of the RNA-seq data were confirmed.

## Discussion

The catalysis of GSH with GST was considered as the first step of detoxifying the fish body from MC^41, 42^. By semi-quantitative RT-PCR, the expression of GST in the liver tissues of silver carps feeding for 2, 4, and 6 d in the two kinds of waters (1.3 × 10^8^ cell/L) was detected. The relative expression of GST in the experimental group on 6 d was remarkably down-regulated. Furthermore, the specimens at the time point were analyzed in subsequent experiments for genes that were initiated under the presence of MC. Preliminary studies on MC-induced gene expression changes in fish were previously conducted. Qu *et al*. (2011) adopted the suppression subtractive hybridization (SSH) on gene expressions in silver carps injected intraperitoneally with MC-LR, and found that 75 specific genes and 38 known highly functional homologous genes, involving immune and cellular transport and metabolism^43^. Cui *et al*. (2011) screened genes in bighead carps induced by MC-LR and found that the involved pathways were associated with energy metabolism, immunity, and apoptosis, while some genes were associated with cytoskeleton, transport, and signal transduction^44^. Consequently, more accurate and in-depth studies were carried out using high-throughput sequencing platforms.

Using Illumina HiSeq4000, we subjected the specimens of the experimental and control groups on 6 d to RNA-seq and thus produced 105,379 unigenes. According to the GO classification statistics, the majority of genes were involved in cellular macromolecule metabolic process (5,378 genes), organic cyclic compound metabolic process (4,586 genes), and cellular aromatic compound metabolic process (4,445 genes). These findings indicated that all of the related genes contributed to the metabolic process of MC in fish. Based on the KEGG pathway analysis, most of the actively expressed pathways were associated with diseases, thus indicating that MCs entering the fish body potentially produce toxic effects. Through the expression difference analysis, 143 were significantly differentiated genes, 82 of which were markedly up-regulated genes, and 61 were remarkably down-regulated genes. GST is a phase II enzyme of the detoxification processes implicated in the conjugation of MCs with GSH^45^. In the present study, the expression of GST was remarkably down-regulated, and the results were consistent with RT-PCR. Gene regulation is a very complicated process, and antioxidant system is a positive role in response to *M. aeruginosa*, which has been verified by multiple levels from biochemical level, translation level to gene expression^26–28^. However, in this study, superoxide dismutase (SOD), catalase (CAT), and glutathione peroxidase (GPx), belonging to major enzymes of the antioxidant system, regulate excess ROS to avoid oxidative damages, as revealed by RNA-seq^46^, their expressions did not show significant changes, indicating the antioxidant system in the experimental fish can act as the control fish during a short exposure period. Cytochrome P450 2k belongs to Phase I enzymes that can support drug metabolism and exogenous substance metabolism, as well as non-synthetic transformation such as oxidation, reduction, and hydrolysis^47^. Its expression was remarkably reduced, suggesting its important role after MC entered the fish body. As revealed by the GO and KEGG enrichment analysis, many immune-associated terms and pathways were significantly enriched, indicating that MC might lead to fish immune dysfunction. Numerous immune-associated genes were involved in immunologic injuries and were critical for the effects of MC on the liver tissues from silver carps and may contribute in resisting MC. The RNA-seq results of silver carp liver tissues injected intraperitoneally with MC-LR were compared^43^. Genes annotated to the COG/GO terms in the RNA-seq analysis on liver tissues from silver carps feeding in the waters containing *M. aeruginosa* and injected intraperitoneally with MC-LR were basically consistent with those to the KEGG terms. The top 18 of the 20 most active KEGG pathways involved were also consistent: pathways in cancer, PI3K-Akt signaling pathway, focal adhesion, proteoglycans in cancer, HTLV−I infection, MAPK signaling pathway, rap1 signaling pathway, cAMP signaling pathway, regulation of actin cytoskeleton, calcium signaling pathway, endocytosis, Huntington’s disease, cGMP−PKG signaling pathway, oxytocin signaling pathway, Alzheimer’s disease, ras signaling pathway, viral carcinogenesis, and platelet activation pathways. Only the pathways associated with adrenergic signaling in cardiomyocytes and tuberculosis were replaced with those associated with protein processing in endoplasmic reticulum and platelet activation. Furthermore, GO and KEGG analysis on DEGs many pathways associated with immune and diseases were significantly enriched, indicating that under the actions of the two *M. aeruginosa* variants, the effects of MC after entering liver tissues were basically the same, causing toxic effects on liver tissues and immune responses. In addition, the two types might have the same detoxifying and antitoxic mechanisms of MC in fish under different actions.

After the conjoint analysis on RNA-seq and miRNA-seq data, four candidate genes, namely, *CABZ01088134.1*, *steap4*, *Cyp7a1*, and *PPP1R3G*, were initially selected. These genes could play critical roles in the intoxicating, detoxifying, and antitoxic mechanisms of MC in fish. Compared with that of the control group, the expression levels of *steap4* and *CABZ01088134.1* were up-regulated and the expression levels of the corresponding regulatory miRNAs were down-regulated. By contrast, the expression levels of *Cyp7a1* and *PPP1R3G* were down-regulated and the expression levels of the corresponding regulatory miRNAs were up-regulated in the experimental group. Additionally, the RNA-seq data were consistent with the qPCR results. Thus, the accuracy of the sequencing technique was confirmed.

Metalloreductase STEAP4 (*steap4*) is a member of the family of the six transmembrane epitope antigens of prostate (*STAMP*). *Steap4* exhibits Fe^3+^-Cu^2+^ reductase activity and plays an important role in Fe^3+^ and Cu^2+^ adsorption^48^. Wellen *et al*. (2007) drew a conclusion that *Steap4* also integrates inflammation and metabolic responses and participates in metabolic homeostasis^49^. The GO terms included binding, catalytic activity, cellular process, single-organism process, and metabolic process, and these terms participated in pyrimidine metabolism, thyroid hormone synthesis, drug metabolism, other enzyme metabolic pathways, and other KEGG pathways. These pathways can be regulated by miRNAs, such as dre-miR-144–5p, 5_37929, and dre-miR-144–3p. The absence of *steap4* in adipocytes may lead to inflammatory response disorders in acute inflammatory conditions. *steap4* integrated inflammatory and metabolic responses, thus playing a part in the metabolic homeostasis in the system^49^. Expression analysis revealed that the expression levels of *steap4* on 2, 4, and 6 d in the experimental group were markedly up-regulated, suggesting that *steap4* may play an important role in inflammatory reaction, metabolic response, and systematic homeostasis in the presence of MC in fish.

Trans-2-enoyl-CoA reductase, mitochondrial-like (*CABZ01088134.1*) is a fatty acyl-CoA elongase for long-chain fatty acid synthesis, which involves the synthesis of glyceride and biofilm, and plays a significant part in growth, development, external environment adaptation, and other processes. The GO term of *CABZ01088134.1* involved membrane part, cellular process, single organism process, and establishment of localization took part in metabolic pathways, and fatty acid metabolism KEGG pathway and might be regulated by miRNAs, such as dre-miR-9-5p and 7_42134. Enoyl-CoA involved in the oxidation cycle of mitochondrial β is a substrate for the second stage of oxidation cycle^50^. Li (2010) proposed that the oxidation of mitochondrial β-fatty acid in nematodes provides the main mechanism of fatty acid decomposition in the body to produce high amounts of energy for the body, while some of the derivatives function as hormones and intracellular messengers^51^. In the present experiment, *CABZ01088134.1* expression was up-regulated as the action time of MC increased and implicated in the metabolic process of MC.

Cholesterol 7-alpha-monooxygenase (*Cyp7a1*), also known as cholesterol 7α-hydroxylase, cholesterol 7α-monooxygenase, or cytochrome P450 7A1 enzyme, belongs to the liver-specific microsomal cytochrome P450 enzyme system and catalyzes the breakdown of cholesterol in the liver into bile acids. Du *et al*. (2013) put forward that the substance also serves as the rate-limiting enzyme in the reaction^52^. Its expression and activity are implicated in bile acid synthesis^53^, and in cholesterol homeostasis maintenance^54^. *Cyp7a1* involved binding, catalytic activity, membrane part, organelles and other GO terms; and took part in metabolic pathways, bile secretion, PPAR signaling pathway and other KEGG pathways. It may be regulated by miRNAs such as dre-miR-489 and 17_15861. Feingold *et al*. (1996) suggested, endotoxin, tumor necrosis factor α (*TNF-α*), and interleukin-1 could lower the mRNA expression of *Cyp7A1* in hamsters and reduce the ability of the liver to treat cholesterol^55^. In the experimental group, the expression of *Cyp7a1* was remarkably down-regulated since 2 d and then maintained at a relatively low level. In general, hydrophilic MC does not easily pass directly through cells. Eriksson *et al*. (1990) revealed that the transport system of bile acid in fish is essential for the transport of MC to the liver^56^. Thus, in the present experiment, the down-regulation of *Cyp7a1* might reduce the ability of the liver to decompose cholesterol and the transport of MC with bile acid to the liver.

Protein phosphatase 1 regulatory subunit 3G-like (*PPP1R3G*) is a regulatory subunit of protein phosphatase 1 (PP1), which is a class of enzymes that catalyze the dephosphorylation of phosphorylated protein molecules. This enzyme is associated with protein kinase and implicated in a system of switching protein activities between phosphorylation and dephosphorylation. *PPP1R3G* may be regulated by miRNAs, such as dre-miR-129-1-3p, 17_15759, and dre-miR-19b-5p. The regulatory subunit did not trigger a catalytic activity. However, its conformation changed by binding to an effector and thus affected the catalytic subunit activity. MC entering the fish body strongly inhibits PP1 and PP2A, as confirmed by Bagu *et al*.^57^. Conducting crystal structure analysis, Maynes *et al*. (2001) found that MC uses the bonds of its catalytic subunit with PP1 and PP2A as targets for attack^58^. In the present experiment, *PPP1R3G* expression was occasionally down-regulated, suggesting that MC might affect the activity of the catalytic subunit.

## Conclusions

In the present experiment, the expression levels of four genes, namely, *CABZ01088134.1*, *Steap4*, *Cyp7a1*, and *PPP1R3G*, in the presence of MC were analyzed. These genes were initiated in early stages and were implicated in the toxic, detoxifying, and antitoxic mechanisms of MC in fish. RNA-seq and miRNA-seq analysis of the liver tissues of *M. aeruginosa*-treated silver carps provided valuable data regarding these mechanisms in fish, and these data could be served as a good reference for further studies on *M. aeruginosa* toxicity.

## Methods

### Fish and algae for experiment

Silver carps used in the present experiment were provided by a mariculture base of Shanghai Ocean University and were in good health, with the average body weight of 105.5 ± 11.2 g (n = 50). The handling and treatment of experimental fish were conducted in accordance with the guidelines set by the Institutional Animal Care and Use Committee (IACUC) of Shanghai Ocean University (SHOU), and this research was approved by the IACUC of SHOU, Shanghai, China. Toxic (strain: FACHB-905) and non-toxic *M. aeruginosa* (strain: FACHB-469) species were provided by the Wildlife Germplasm Library of Freshwater Algae of the Chinese Academy of Sciences. BG-11 algae culture medium was used for enlargement culturing in the incubator. Culture temperature: 26 °C; light to dark ratio (h/h): 12:12; and light intensity: 6600 Lx. The density of algal cells was determined by using hemocytometer, while the MC toxin type and content were detected by high-performance liquid chromatography–mass spectrometry (HPLC-MS)^59^.

### Treatment experiment and collection of the liver tissue specimens and extraction of total RNA

Silver carps were acclimated for three days in a full aeration tap water, without feeding. Silver carps of the experimental group were cultured in waters containing toxic *M. aeruginosa* (Density: 1.3 × 10^8^ cell/L, this concentration can be found in some inland waters with harmful algal blooms), while those of the control group were cultured in waters containing non-toxic *M. aeruginosa* (Density: 1.3 × 10^8^ cell/L). Specimens were collected every 6 h. The algal density was observed using hemocytometer under a microscope. A right amount of algal culture solution was added after calculation to maintain the density of each of the two *M. aeruginosa* variants in waters at the level of 1.3 × 10^8^ cell/L. Three silver carps of each of the experimental and the control groups after exposure for 2, 4 and 6 d were randomly collected with liver tissue specimens. Total RNA was isolated from liver tissues using a miRNeasy kit (Takara, Dalian, China) according to the manufacturer’s instruction. The RNA quality was determined using an Agilent 2100 Bioanalyzer (Agilent, Germany). Specimens after total RNA extractions were dissolved in 50 μL 0.1% DEPC-ddH_2_O. The extracted total RNA was stored at −80 °C after OD value and concentration were determined.

### Determining the expression of GST by semi-quantitative RT-PCR

With β-actin as the reference gene^60–62^, the expression of GST was detected by semi-quantitative RT-PCR at the three time points, namely after exposure for 2, 4 and 6 d, in the two kinds of waters. The earliest time point at which GST was initiated in presence of MC was selected for follow-up experiment. The primer sequences used in semi-quantitative RT-PCR analysis were listed in Table 1.

### RNA-seq and de-novo assembly

The raw data acquired from the sequencing was screened using SeqPrep and Sickle softwares to obtain a clean data and ensure smooth progress of the follow-up analysis. Contig and singleton were acquired from all clean data using Trinity software after de-novo assembly.

### Gene annotations

NCBI non-redundant protein library (NCBI_NR, http://www.ncbi.nlm.nih.gov/), String (http://string-db.org/), Swissprot (http://www.ebi.ac.uk/uniprot/), Clusters of Orthologous groups of proteins (COG, http://www.ncbi.nlm.nih.gov/COG/), Gene Ontology (GO, http://www.geneontology.org/) and Kyoto Encyclopedia of Genes and Genomes (KEGG, http://www.genome.jp/kegg/) were compared by using BlastX. Thus, the corresponding annotations were obtained.

### miRNA-seq

The quality of raw reads acquired from the small RNA sequencing was controlled by using SeqPrep and Sickle to obtain clean reads. Blast was then used to annotate the acquired small RNAs according to the Rfam library, where non-miRNA sequences were eliminated. The clean reads with a length of 18–30 nt were blasted against the GenBank noncoding RNA database and the Rfam database to annotate tRNA, rRNA, snRNA, repeat associate small RNA, and other ncRNA sequences. Since the whole-genome sequence of *H. molitrix* is unknown, the remaining reads were used to map to the zebrafish *Danio rerio* genome. Then the mapped reads were aligned with miRNAs of zebrafish and the matched sequences were identified as known miRNAs^63^.

### Expressions of known and novel miRNAs and prediction of their target genes

Small RNA sequences with eliminated rRNA, scRNA, snoRNA, snRNA, and tRNA sequences were compared with miRNA precursors and mature sequences of the species in the miRBase library by using Bowtie, RNAfold, and miRDeep2. Relevant statistics was mapped to the number of mature sequences and secondary structures were predicted. Novel miRNAs were predicted by the pre-miRNA hairpin by utilizing miRDeep2, Randfold, Bowtie, and NAfold. Overlapping sequences were used to form longer sequences according to their alignments to known precursor sequences in the miRBase^64^. Novel miRNAs were then identified on the basis of the results combined with Dicer restriction site and energy value. The target genes of all known and novel miRNAs were predicted with Miranda, and annotations to the target genes were mapped to zebrafish (*Danio rerio*) miRNAs correctly^63^.

### Conjoint analysis on the RNA-seq and miRNA-seq data and real-time fluorescence quantitative PCR

The ortholog genes were classified by blast-based method, while the target genes of miRNAs were matched with the homologous genes of the differentially expressed genes (DEGs) in transcriptome sequencing^65^. The fold change higher than 2 and a p-value < 0.05 were the criteria to select differentially-expressed miRNAs. Through qPCR, the expressions of *steap4*, *Cyp7a1*, *PPP1R3G*, and *CABZ01088134.1* were detected on 2, 4, and 6 d and the accuracy of the RNA-seq data was validated. The primer sequences used in quantitative real-time PCR analysis were listed in Table 1.

### Statistical analysis

Statistical analyses of the data were performed using SPSS 17.0. Sample size for the experimental/control group each sampling time was 3, and the technical replicate for each sample was 2. All values were presented as the mean ± standard deviation (SD). Normality and homogeneity of variances of the data were checked by Shapiro-Wilk’s W test and Levene’s test, respectively. The data on differentially expressed genes or miRNAs were statistically analyzed based on the method of Audic and Claverie (1997)^66^. Significant differences between toxic algal treatment and control were determined using a Student t test. Significant differences were denoted by p < 0.05 and extremely significant differences were indicated by p < 0.01.

**Table1:** Primer sequences used in semi-quantitative RT-PCR and quantitative real-time PCR analysis.

Primer	Sequence	Product size (bp)
GST-F	5′-AGAACGGGCTTTGATTGAC-3′	267
GST-R	5′-AAGGTTGACAGTATTGTAGGGA-3′	
ACT^1^-F	5′-ATTGCCGCACTGGTTGTT-3′	340
ACT^1^-R	5′-TTTCCCTGTTGGCTTTGG-3′	
CABZ01088134.1-F	5′- TGAACCCCTGCACTGCCTAC-3′	158
CABZ01088134.1-R	5′- CTGTCTCTGATGATGTTGATGGTCT-3′	
steap4-F	5′- CCTTCAACACCATCTCTGCCTG-3′	146
steap4-R	5′- GCCTCTGTCCAGGACTGTGAAAC-3′	
*Cyp7a1*-F	5′- ATTCTCCTACCACGCCGTCA-3′	137
*Cyp7a1*-R	5′- CTGATGCAGATTTTCAGTGGTGTAAC-3′	
PPP1R3G-F	5′-GAAAGACAGGAGAAGAGCCAAGT-3′	212
PPP1R3G-R	5′-GGTCCTTATGAAGAGGGAAACTC-3′	
ACT^2^-F	5′- TGAGAGGTTCAGGTGCCCAG-3′	278
ACT^2^-R	5′- TGTCAGCAATGCCAGGGTAC-3′	

## Electronic supplementary material

Supplementary information
