# Supplementary material for: Effects of toxic Microcystis aeruginosa on the silver carp Hypophthalmichtys molitrix revealed by hepatic RNA-seq and miRNA-seq
Source: Sci Rep. 2017 Sep 5;7:10456. doi: 10.1038/s41598-017-10335-9 (PMC5585339; doi:10.1038/s41598-017-10335-9)
Supplement: Supplementary file 1 — Supplementary information [file 41598_2017_10335_MOESM1_ESM.doc]

Supplementary Information

**Article title**: Effects of toxic *Microcystis aeruginosa* on the silver carp *Hypophthalmichtys molitrix* revealed by hepatic RNA-seq and miRNA-seq

**Authors**: Menghong Hu, Xiancheng Qu, Lisha Pan, Chunxue Fu, Peixuan Jia, Qigen Liu, Youji Wang.

The following Supporting Information is available for this article:

The RNA/miRNA data availability from NCBI database

**The RNA/miRNA data availability from NCBI database**

bioproject : PRJNA325567 Registration No：SRP090341

biosample: SAMN05267366 Registration No：SRS1710466

There were four RNA data and four miRNA data respectively. Since the original data of RNA in the experimental group were uploaded twice on the sixth day, the amount of accession number from NCBI is 9.

1. The original RNA data of Sample 1 in the experimental group on the sixth day

SRA number：SRR4292161 File 6111_S12_L005_R2_001.fastq.gz

SRA number：SRR4292178 File 6111_S12_L005_R1_001.fastq.gz

2. The original RNA data of Sample 2 in the experimental group on the sixth day

SRA number：SRR4294186

File ：6112-3_S9_L005_R1_001.fastq.gz

6112-3_S9_L005_R2_001.fastq.gz

3. The original RNA data of Sample 1 in the control group on the sixth day

SRA number：SRR4294640

File ：6121_S11_L005_R1_001.fastq.gz

6121_S11_L005_R2_001.fastq.gz

4. The original RNA data of Sample 2 in the control group on the sixth day

SRA number：SRR4296545

File ：6122-3_S10_L005_R1_001.fastq.gz

6122-3_S10_L005_R2_001.fastq.gz

5. The original miRNA data of Sample 1 in the experimental group on the sixth day

SRA number：SRR4301760

File ：6111.fq.gz

6. The original miRNA data of Sample 2 in the experimental group on the sixth day

SRA number：SRR4301761

File ：6112_3.fq.gz

7. The original miRNA data of Sample 1 in the control group on the sixth day

SRA number：SRR4301762

File ：6121.fq.gz

8. The original miRNA data of Sample 2 in the control group on the sixth day

SRA number：SRR4301763

File ：6122_3.fq.gz
